# Morphological Diversity of Pretibial Myxedema and Its Mechanism of Evolving Process and Outcome: A Retrospective Study of 216 Cases

**DOI:** 10.1155/2016/2652174

**Published:** 2016-08-25

**Authors:** Changgui Lan, Yi Wang, Xia Zeng, Jing Zhao, Xiaoxi Zou

**Affiliations:** Department of Dermatology, China National Nuclear Corporation (CNNC) 416 Hospital, No. 4 Er Huan Lu Bei Si Duan, Chengdu, Sichuan 610051, China

## Abstract

*Background*. Pretibial myxedema (PTM) is a rare dermopathy. The morphologic features and mechanism of its evolving process are not reported in large case series.* Methods*. 216 cases with PTM were retrospectively reviewed to analyze demographics, history, lesional morphology and its evolving process, histopathology and immunohistochemistry, serum TRAb levels, treatment, and outcome.* Results*. First appearing lesions evolved into 6 variants that were correlated with serum TRAb levels. Subvariants were caused by different kinds and frequencies of local trauma. The evolving process could be classified into 4 stages that were correlated with serum TRAb levels and perivascular infiltration of CD8+ and CD4+ lymphocytes. Serum TRAb levels at remission and in nonrecurred cases became lower than those before therapy and in recurred cases, respectively, but increased when PTM relapsed. TRAb level in nodule variant went down invariably with the extension of course and its autoimmune activity had a trend to stop but in other 5 variants TRAb levels fluctuated. Their autoimmune activities had no trends to stop and clinically worsen through intermittent repeats of active and stable stages.* Conclusions*. In the chronic course of PTM, nodule variant is self-limited and other 5 variants are not self-limited. PTM needed early treatment to avoid severe variants.

## 1. Introduction

Pretibial myxedema (PTM), or localized myxedema, more accurately, a localized thyroid-associated dermopathy, first reported by Hektoen in 1895, is characterized by local thickening and remodeling of the skin and always associated with a group of autoimmune thyroid diseases [1–3]. While it is commonly located on the pretibial area, actually, it could occur on the skin of all body sites. It is relatively rare in clinical practice because its prevalence in Grave's disease is only 1.5–1.7% [4, 5]. Up to now, it has been widely accepted that most cases of PTM are inconspicuous and tend to resolve spontaneously [6–11]. Clinically PTM is generally thought to be only of cosmetic importance. Only severe patients acquire treatment but response to treatment is not optimal in many instances although therapeutic modalities for PTM have been reported and local glucocorticoid treatment has been accepted by some endocrinologists [12]. In addition, it is often prone to be overshadowed by more importance of ophthalmopathy. So the dermopathy has long been ignored by patients and doctors. However, the experience from our patients seems to differ from widely accepted one, especially the evolving process and outcome of PTM. Though isolated cases of its clinical features have been reported and its variants have also been mentioned in PubMed database, detailed and comprehensive study of morphological features and its evolvement have not been described in a large case series. Likewise, there have been no reports about the study of mechanism of evolving process of PTM variants. Therefore, the retrospective study in a large series of 216 cases was performed to reveal the morphologic feature, the mechanism of its evolving process, and outcome of PTM.

## 2. Patients and Methods

### 2.1. Patients

Cases with PTM diagnosed and treated at Department of Dermatology in CNNC 416 Hospital from March 11, 2012, to October 21, 2015, were retrospectively reviewed on the basis of diagnostic criteria, inclusion and exclusion of the study.

In the study, diagnostic criteria of PTM included (1) autoimmune thyroid diseases or its history; (2) nonpitting swelling and thickening of local skin; and (3) mucinous degeneration and positive Alcian blue staining that were the most marked features of histopathology, (4) apart from mucinous degeneration caused by other primary and second cutaneous mucinoses.

Exclusive criteria were lack of one record of the followings: demographic data; history; morphologic description of lesions; histopathological examination; and serum thyrotropin receptor antibody (TRAb).

The eligible case was to meet the diagnostic criteria but not excluded from the exclusive criteria.

### 2.2. Classification of PTM Variants and Subvariants

Clinical forms of PTM lesions were called variants and different forms in a variant were named subvariants. The variants of PTM lesions were classified according to the definition of primary lesions in Andrews' disease of the skin-clinical dermatology [13] and description of PTM variants in textbook of dermatology [14]. Subvariants were classified according to the surface contours of a variant.

### 2.3. Assessment of Evolving Stages of Pretibial Myxedema

In the evolving process of PTM, its stages were assessed on the basis of 4 clinical signs of lesions: size and thickness of lesions, new lesions, erythema (redness), and fibrosis [15, 16]. The limit of time was 1 month.

### 2.4. Protocols of Treatment, Outcome Measures, and Follow-Up

**Protocol 1 .**

Intralesionally multipoint injection of triamcinolone acetonide acetate was adopted from Lan's report [17]. The dosage of triamcinolone acetonide acetate was 50 mg/5 mL once a week and treatment was performed to swelling lesions disappearing.

**Protocol 2 .**

The method was also multipoint injection of the above-mentioned steroid. The dosage was 50 mg/5 mL once a week for 8 weeks and then once a month for 6 months.

**Protocol 3 .**

This protocol was called surgery plus intralesional steroid. It was adopted from Lan's reports [18, 19].

*Outcome Measures*. Responses to therapy were recorded as complete response when the reduction of lesion volume was 100%, partial response when the reduction ≥30%, <100% and no response when the reduction <30%, or no reduction or even increase.

In the 1.5-year follow-up, patients were assessed with outcome measures and serum TRAb levels were measured. Relapses of lesions were treated with Protocol 1.

### 2.5. Measurement of TRAb

Serum TRAb (also called thyroid-stimulating hormone receptor antibody, or TSHR antibody) level was measured with electrochemiluminescence immunoassay (anti-TSHR kits in Elecsys and Cobas e 601 analyzer, Roche Diagnostics GmbH, Germany) [20]. The normal value of serum TRAb level was 0–1.75 IU/L.

### 2.6. Histopathological and Alcian Blue Staining Procedures

Skin biopsies were routinely fixed with 5% formaldehyde, embedded in paraffin wax, and stained with haematoxylin-eosin (HE) and Alcian blue dye at pH 2.5.

### 2.7. Immunohistochemical Procedures

Tissue sections embedded in paraffin wax were deparaffinised and rehydrated by a graded ethyl alcohol series decreasing in concentration ending in water. CD4 and CD8 were immunostained with mouse anti-human CD4 (monoclonal number: SP35) and CD8 (monoclonal number: C8/144B) monoclonal antibodies (Fuzhou Maixin Biotech Co., Ltd.) and streptavidin-peroxidase method.

### 2.8. Statistical Analysis

SPSS Statistics 17.0 software was used to describe demographic, associated dysthyroid and clinical characteristic data. The means of serum TRAb levels were analyzed by one-way ANOVA, paired samples *t*-test, and independent samples *t*-test, respectively. *p* ≤ 0.05 was statistical difference and *p* ≤ 0.01 was significant difference.

## 3. Results

A total of 264 cases with PTM (238 at outpatient and 26 at inpatient) were collected from March 11, 2012, to October 21, 2015. 48 cases were excluded because 5 cases were repeated, 20 cases were lack of clinically complete descriptions, and 23 cases had no skin biopsies. Finally, 216 cases with PTM entered the study.

### 3.1. Demographic and Dysthyroid-Associating Characteristics of Pretibial Myxedema When Diagnosed at Our Department

The demographic and dysthyroid-associating characteristics of 216 cases with PTM were shown in Table 1. 120 cases (56%) were the female and 96 cases (44%) were the male. The age ranged from 17 to 80 years and its average was 46.7 ± 12.2 years. More than a half of patients were Chinese farmer. The occupation also included city resident, worker, cadre, and teacher.

215 (99.5%) cases with PTM were Grave's disease and only one (0.5%) case was Hashimoto thyroiditis. At onset of PTM, 24.54% cases were hyperthyroidism (30 cases simultaneously with hyperthyroidism and 23 cases after therapy of hyperthyroidism), 11.57% were hypothyroidism (1 case with hypothyroidism of Hashimoto thyroiditis and 24 cases after therapy of hyperthyroidism), and 63.89% were euthyroidism (4 cases before hyperthyroidism and 134 cases after therapy of hyperthyroidism). PTM occurred most often (84.26%) after the onset of dysthyroidism and 13.89% was simultaneously with dysthyroidism, occasionally (1.85%) before dysthyroidism. The dysthyroid course was 50.64 ± 46.47 months. Mean time between dysthyroid diagnosis and PTM onset was 27.6 ± 30.8 (1–191) months (2.3 years). Extrathyroid diseases associated with PTM were often exophthalmos (95.4%) and infrequently acropachy (12.96%).

### 3.2. Classification of Variants and Subvariants and Its Morphological Characteristics

Morphological characteristics of the 6 variants and its subvariants were as follows.

**Figure 1:**
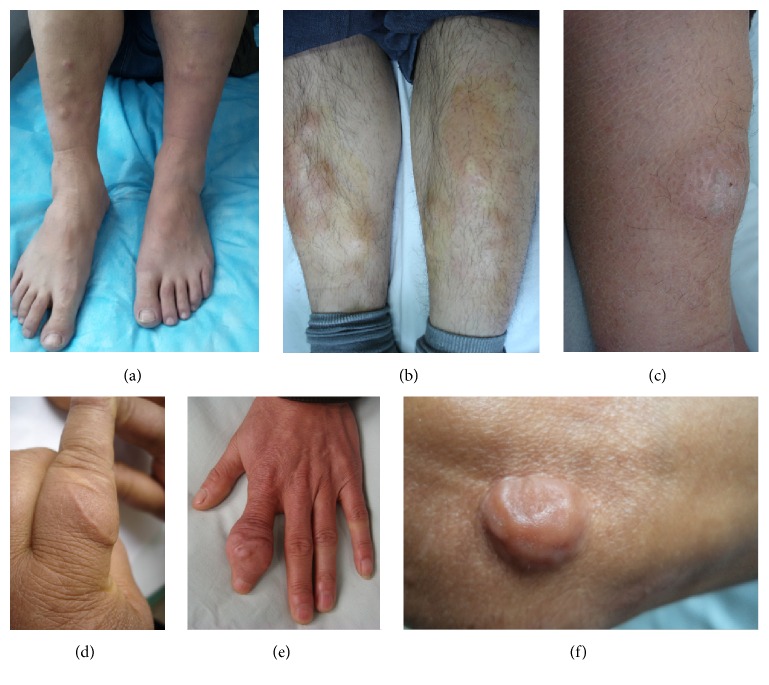
Nodule variant. (a) Multiple skin-color nodules on the extensors of lower legs. (b) Multiple red nodules on the extensors of lower legs. (c) A single scaly and erythematous nodule on the extensor of left lower leg. (d) A single, poorly defined and skin-color nodule at the traumatic scar of left proximal index finger. (e) A single, ill-defined, and skin-color nodule at the scar of left distal index finger. (f) A single, well-circumscribed, and keloid-like nodule at the scar of right elbow.

The nodule variant only had the lesion of nodule. The nodule was a circumscribed, solid lesion with a diameter of less than 2 cm. It could be elevated or not but could be touched in the skin. Sometimes traumatic scars or scales could be visible on the surface of lesions. The color might be red, or skin-colored, or pigmented. Its number might be one or multiple (see Figure 1).

**Figure 2:**
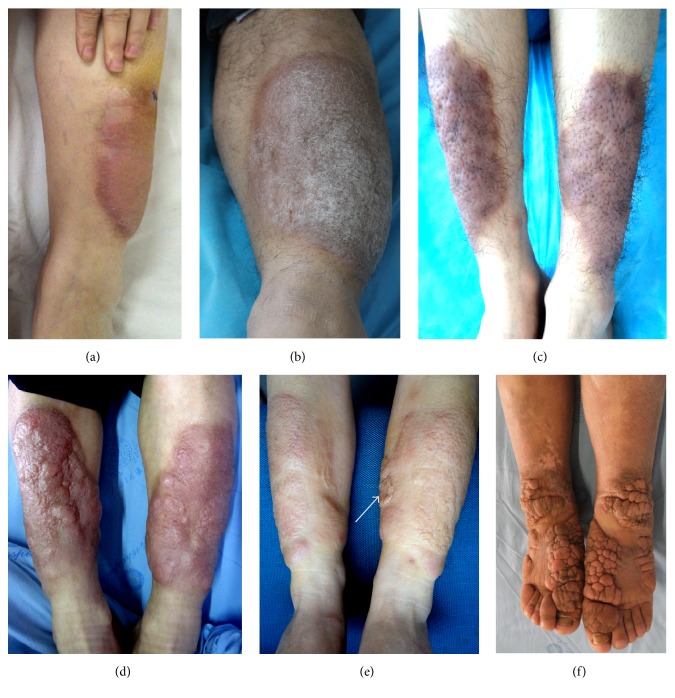
Plaque variant. (a) Well-circumscribed and bright red plaques with smooth appearance at the extensors of lower legs. (b) A single scaly, erythematous plaque at the extensor of left lower leg. (c) Well-circumscribed, pigmented, and orange peel-like plaques with hypertrichosis on the extensors of lower legs. (d) Well-circumscribed, scarlet, and orange peel-like plaques with papule-nodule surface on the extensors of lower legs. (e) Red, orange peel-like plaques on the extensors of lower legs and the arrow indicated a hypertrophic scar. (f) Multiple, skin-color, and fungating plaques on the back of toes and feet and at the flexor aspect of ankles.

The plaque was the only lesion or major lesion of plaque variant. A plaque was a superficial and flat-topped protrusion over the surface of skin. Its diameter was >1 cm. The color might be reddish or pigmented. Its subvariants could be smooth, or scaly, or papule-nodule mixture surface. Multiple round, pileus-like (top parts of mushrooms) nodules on the plaque were defined as fungating subvariant (see Figure 2(f)). Localized hypertrichosis and an orange peel-like appearance were often seen (Figure 2).

**Figure 3:**
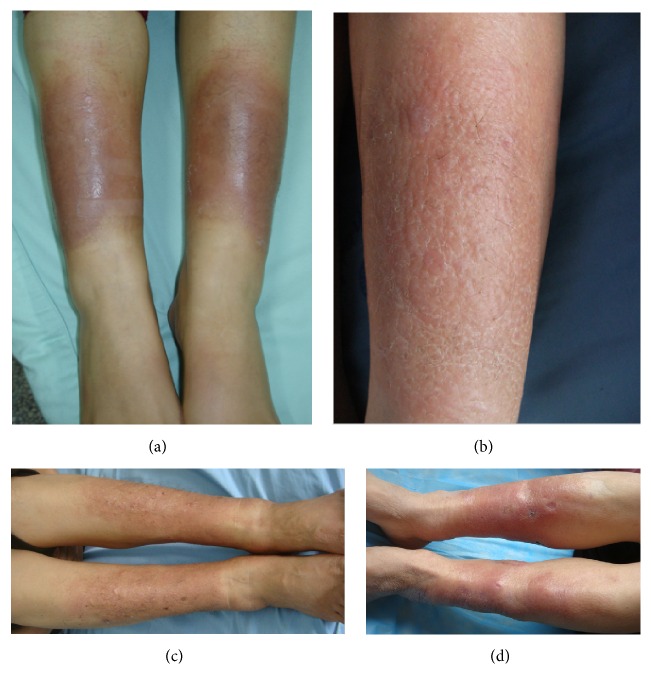
Diffuse swelling variant. (a) Well-circumscribed, erythematous, and nonpitting diffuse swelling (thickening when touched) of skin at the half extensors of lower legs. (b) Scaly, red, irregular circumscribed, and nonpitting diffuse swelling with orange peel-like appearance at lower two-thirds circumference of lower legs. (c) Ill-defined, erythematous, papules-and-bloody-crust scattered, and nonpitting diffuse swelling with local hypertrichosis at the lower two-thirds extensors of lower legs. (d) Scarlet, bloody crust, and nonpitting swelling of skin with atrophic scars at the lower three-fourths circumference of lower legs.

The diffuse swelling variant presented as diffusely cutaneous thickening but not obviously elevated over the surface of skin, with involvement of skin in one-third to three fourths of the lower leg or more. The swelling was hard and nonpitting. Its subvariants might be smooth, or orange peel-like, or hypertrichosis, or scattered papules (Figure 3).

**Figure 4:**
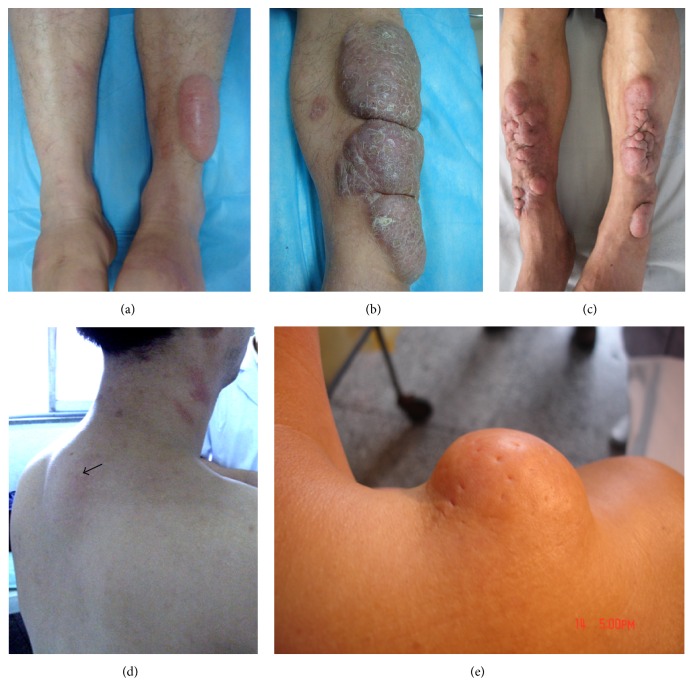
Tumor variant. (a) An olive-like, tense, and erythematous tumor with smooth surface on the extensor of left lower leg. (b) An erythematous, lobulated tumor with scales and crusts on the extensor of left lower leg. (c) Multiple erythematous, dermatofibrosarcoma-protuberans-like tumors, and nodules on the extensors of lower legs. (d) A skin-color, olive-like tumor at the interscapular region. (e) A skin-color, hemisphere tumor with orange peel-like appearance on the right shoulder.

The tumor variant presented as a single or multiple masses. The diameter of a mass was more than 2 cm. Its subvariants could be smooth, or scaly, or lobulated, or orange peel-like appearance. The dermatofibrosarcoma-protuberans-like appearance with multiple, red masses, nodules, and plaques (Figure 4(c)) was defined as the dermatofibrosarcoma-protuberans-like subvariant (Figure 4).

**Figure 5:**
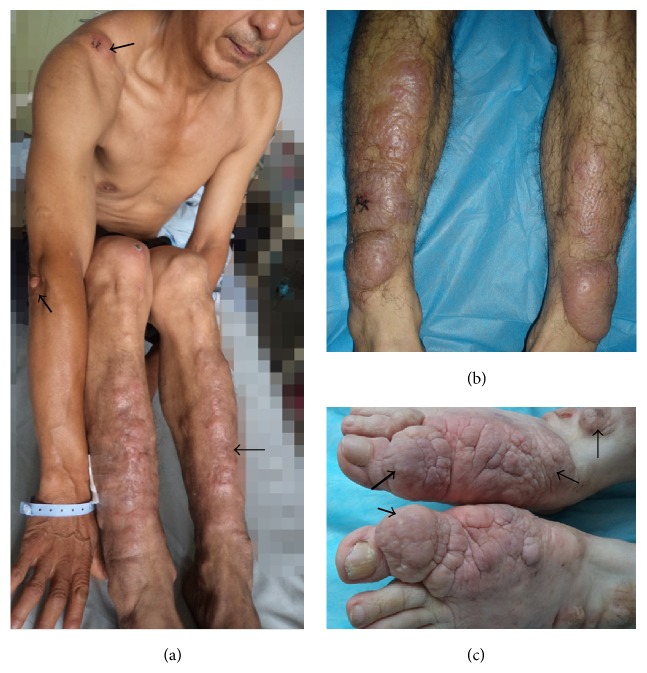
Mixture variant. (a) 4 × 4 cm hemisphere tumor with orange peel-like appearance on the right shoulder, a keloid-like nodule at the extensor of right elbow, and nodules and plaques on the lower two-thirds extensors of lower legs. (b) Red tumors and plaques on the extensors of lower legs. (c) Wide arrows indicated tumors on the first toes; a fine arrow showed red plaques with polypoid appearance on the back of feet and a red nodule at the flexor aspect of right ankle.

The mixture variant manifested as coexistence of two or more of the above-mentioned variants (Figure 5).

**Figure 6:**
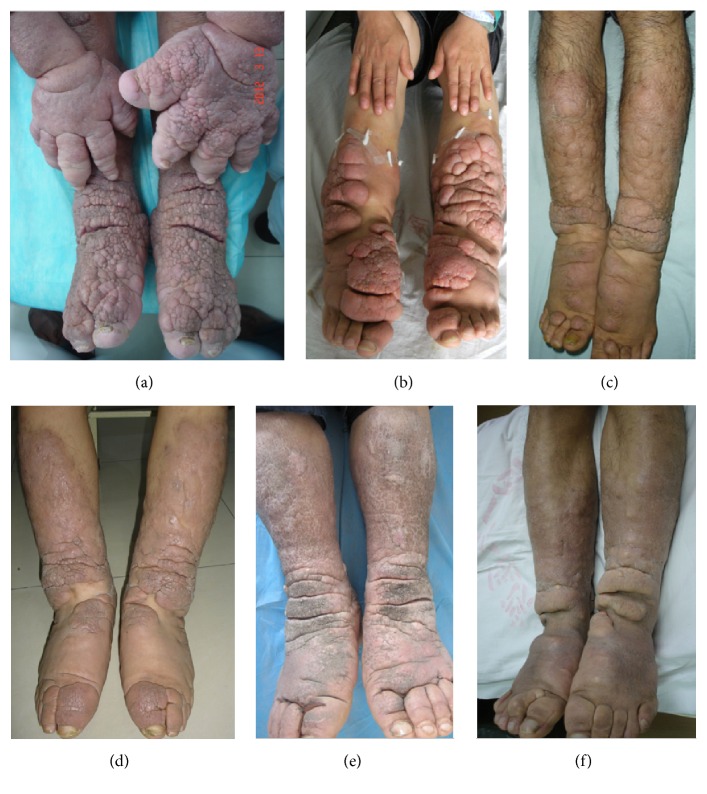
Elephantiasis variant. (a) Elephantiasic legs and hands with hyperpigmented and polypoid appearance. (b) Red, swelling enlargement of lower extremities with nodular, polypoid, fungating, and tumorous appearances from lower one-half of lower legs to toes. (c) Light red, swelling enlargement of lower extremities with nodular, tumorous, and papillary appearance from lower two-thirds of lower legs to toes. (d) Light red, pigmented, nonpitting and swelling enlargement of lower extremities with plaque, papillary, and tumorous appearances from lower legs to toes. (e) Swelling enlargement of lower extremities with hyperpigmented, dry, coarse, papillary, and deep crevice appearance looked extremely like legs of elephants. (f) Pigmented, sclerotic enlargement of lower legs to toes with cerebriform and plaque appearance.

The elephantiasis variant was characterized by dramatically diffuse enlargement of extremities with plaques, nodules, or masses scattered on the smooth or polypoid or papillary surface. Local, polyp-like or granuloma-like protrusions with particles or papules on the surface were called polypoid subvariant. The coarse, uneven surface with hyperkeratosis and superficial fissures, looking like the surface of nipples of mammary gland, was defined as papillary subvariant. The contour appearance with cerebral gyrus and sulcus was defined as cerebriform subvariant in which there were deep depressions or fissures on the surface of lesions (Figure 6).

When diagnosed at our department, the lesions of 216 cases were divided into the nodule (45 cases), plaque (33 cases), diffuse swelling (96 cases), tumor (10 cases), mixture (17 cases), and elephantiasis (15 cases) variants. The nodule variant accounted for 20.8%. 44.5% were diffuse swelling. Tumor, mixture, and elephantiasis occupied 34.7%.

The demographic and dysthyroid-associating characteristics of 6 variants were summarized in Table 1. In the elephantiasis, tumor, and nodule variants, the male were more than the female but in plaque, diffuse, and mixture the female was more than the male. The age in tumor variant was the smallest but in elephantiasis variant was the biggest.

### 3.3. Definition of Evolving Stages and Its Morphological Characteristics

According to 4 clinical signs of lesions and the limited time of 1 month, the morphological characteristics of 4 stages were defined as follows.

**Figure 7:**
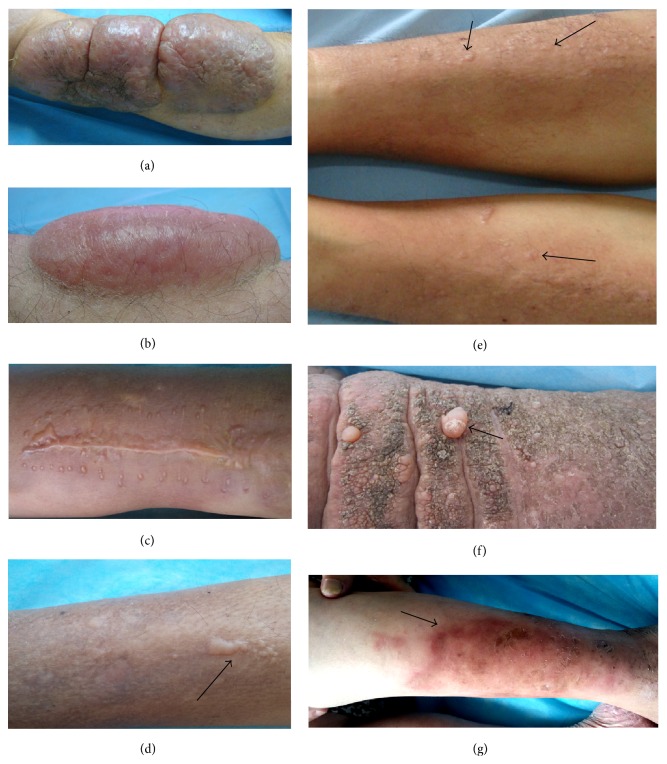
Active stage. (a) Red, crusted (after itching scratch), tense, and semitranslucent appearance of lobulated tumors on the lower leg. (b) Red, tense, and semitranslucent appearance of a smooth tumor on the lower leg. (c) New-formed, hypertrophic-scar-like papules and plaques on the lower leg when PTM was active at the healing wound site after trauma and suture. (d) An arrow indicated new-formed papules and plaques on the active lesions of PTM on the lower leg. (e) Arrows indicated new-formed, red papules on the active lesions of diffuse swelling variant at the lower legs. (f) An arrow indicated tense, pearl-like papule and nodule on the active elephantiasis with dry, papillary appearance on the lower legs. (g) An arrow indicated irregular, scarlet, and swelling erythema on the legs when PTM recurred.

At active stage, the lesion manifested as swelling erythema, or new papule, or new plaque, or new nodule, or enlargement (infiltration into its surrounding skin or protrusion over the surface of the skin or both) in 1 month (Figure 7).

**Figure 8:**
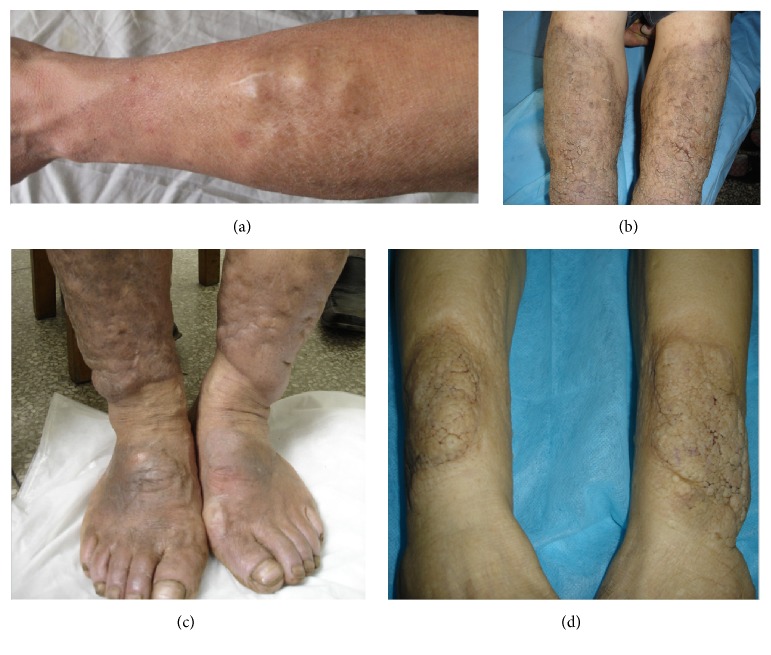
Stable stage. (a) Skin-colored nodules on the legs were kept at the same size for more than 3 months. (b) Well-circumscribed, pigmented, and papillary elephantiasis at stable stage on the legs. (c) Pigmented elephantiasis scattered with pigmented nodules and without erythema at stable stage on the lower extremities. (d) Well-circumscribed, skin-colored plaques with papillary appearance at stable stage on the legs.

At stable stage, PTM presented as skin-colored or pigmented lesions without active features and no expansion over 1 month (Figure 8).

**Figure 9:**
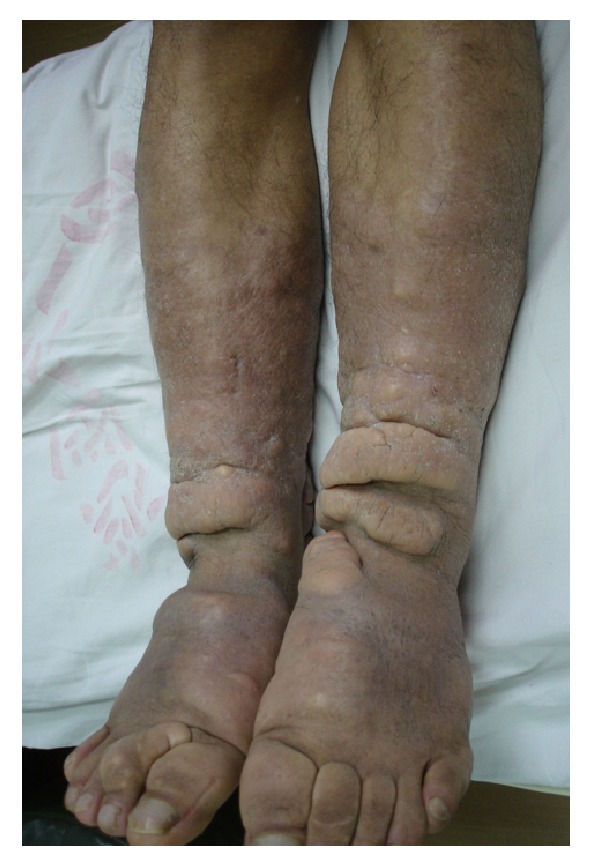
Sclerotic stage. Pigmented, woody hard lesions of elephantiasis without any active signs.

At sclerotic stage, PTM presented as desiccated, pigmented, thickened, and hardened lesions without active features (Figure 9).

**Figure 10:**
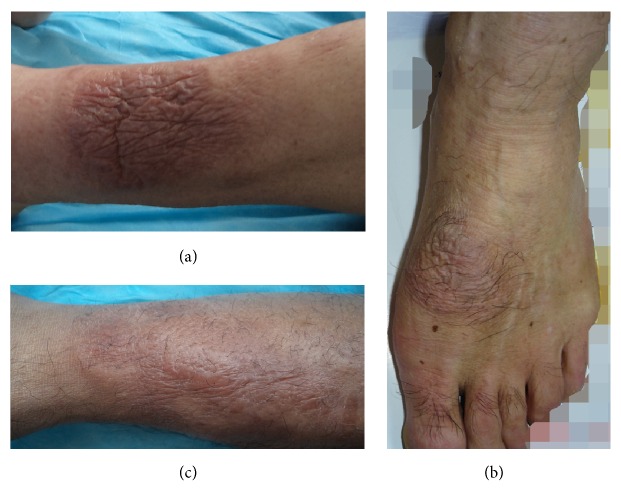
Receding stage. (a) The erythematous, flat lesion with shrinking wrinkles at receding stage on the leg. (b) The well-circumscribed, erythematous, and flat lesion with shrinking wrinkles and hypertrichosis at receding stage on the dorsum of a foot. (c) The ill-defined, erythematous lesion with shrinking wrinkles and tense swelling plaques at receding stage on the leg.

At receding stage, the lesion was receding. Its diameter decreased or its volume became smaller and the circumference of lesions shrank into the center. The depth of thickening skin became superficial, or protrusions of nodules or plaques became flat (Figure 10).

When first diagnosed at our department, the lesions of 216 cases were divided into 4 stages including active, stable, sclerotic, and receding stages. 185 cases were at active stage, 17 cases at stable stage, 2 cases at sclerotic stage, and 12 cases at receding stage. In 45 cases with nodule variant, 36 cases were at active stage, 5 cases at stable stage, and 4 cases at receding stage. In 171 cases with other 5 variants, 149 cases were at active stage, 12 cases at stable stage, 2 cases at sclerotic stage, and 8 cases at receding. In the 12 cases with receding stage, 11 cases received intralesional steroid therapy in other hospitals and one case receded spontaneously without any therapy. The stage distributions of 216 cases in 6 variants were summarized in Table 2.

### 3.4. Clinical Manifestations of Pretibial Myxedema When and before Diagnosed at Our Department

When diagnosed at our department, symptoms and signs of 216 PTM cases were summarized in Table 2. The course of PTM was from 20 days (0.67 months) to 16 years (192 months). Almost half of them had a more than 1 year course. About one-third of cases (27.3%) had more than 2 years. 14.4% had more than 5 years. 53.7% were associated with pruritus, 44.91% with hyperhidrosis, 30.09% with hypertrichosis, and 50% with orange peel-like appearance. The lesions of 216 cases consisted of 20.83% nodule, 15.28% plaque, 44.45% diffuse swelling, 7.87% mixture, tumor (4.63%), and 6.94% elephantiasis. 85.7% were active, 7.9% stable, and 5.5% receding, with only 0.9% sclerotic. All of 216 cases had involvements of lower legs. Feet and toes were involved in 65 cases. Only 3 cases had involvements of fingers, 2 cases of upper extremities, 1 case of right shoulder, and 1 case of interscapular region. 30.09% had local wound history. Almost half of 216 cases (104/216) had traumatic scars on their lesions. There were statistical differences of local injury history and traumatic scars among the 6 variants (*p* = 0.021 and *p* = 0.036). The elephantiasis had the highest percentage (66.7% and 80%). The nodule had the lowest percentage (26.7% and 31.1%). In diffuse swelling, mixture, and elephantiasis variants, the cases with scars were more than the cases without scars. 72 cases had relapse histories after remission by intralesional glucocorticoid. 143 cases had no remission histories without any therapy and one case receded spontaneously without any therapy. There was no statistical difference of PTM relapse after intralesional steroid among 6 variants.

### 3.5. Evolving Process of PTM Lesions

Where did the 6 variants come from? At the onset of PTM, the first appeared lesions were nodules (118 cases, 54.63%), swelling erythema (96 cases, 44.44%), and papules (2 cases, 0.93%), respectively. However, when diagnosed at our department, in 118 cases, 45 cases still stayed in the nodule variant and 73 cases evolved into other 5 variants. The swelling erythema evolved into 5 variants except the nodule variant. The papule had only 2 cases and progressed to mixture and elephantiasis, respectively (see Table 3).

How did the first appearing lesions clinically evolve to 6 variants? When diagnosed at our department, 216 cases were divided into 185 cases at active stage, 17 cases at stable stage, 2 cases at sclerotic stage, and 12 cases at receding stage. To retrospect the histories of 216 cases before being diagnosed at our department, 44 cases in nodule variant underwent intermittent periods of active and stable stages after their onsets; although they still stayed at nodule variant, 1 case in nodule variant was first at active stage and then stabilized, finally receding spontaneously. 171 cases in other 5 variants obviously worsened through the intermittent periods of active and stable stages. So the evolvement of PTM lesions underwent different evolving stages from first appearing lesions to 6 variants.

### 3.6. Variants Mainly Determined by Autoimmunity but Subvariants by Local Injury

Why the variants and subvariants were formed? The relationship between variants of PTM and serum TRAb levels had been investigated in the 216 cases with PTM. Means of TRAb levels in 6 variants were shown in Table 4. There was statistical difference of serum TRAb levels among 6 variants (*F* = 2.817, *p* = 0.017). When performing* Post Hoc* Tests, serum TRAb levels of nodule and tumor variants (66.61 ± 121.70 and 48.61 ± 31.26 IU/L) were lower than those (196.22 ± 298.39 IU/L and 136.48 ± 142.32 IU/L) of elephantiasis and diffuse swelling, respectively. Serum TRAb levels of plaque and mixture were 103.77 ± 102.79 and 122.27 ± 129.47 IU/L, respectively. These data demonstrated the severity of PTM was positively correlated with serum TRAb levels.

In 171 cases with 5 variants except nodule variant, 91 cases had histories of different kinds of local injuries. These local injuries happened or repeatedly happened 1 to 2 months before appearance of papillary or polypoid or mixture of nodules and plaques lesions. These injuries included scratch, surgery, open trauma, and close trauma of skin. 80 cases had smooth surface of lesions without any history of local injury or scratch. These showed that the subvariants in each variant could be caused by different frequencies and kinds of local injuries.

### 3.7. Correlation of TRAb Levels with Evolving Stages

Serum TRAb levels of 4 stages in 6 variants were showed in Table 4. Totally, there was significant difference of serum TRAb levels among 4 stages (*F* = 4.279, *p* = 0.006). The serum TRAb level was the highest at active stage and the lowest at stable stage. The level of serum TRAb at active stage was higher than those of other 3 stages.

At active stage, there were 185 cases including 36 cases in nodule, 30 cases in plaque, 84 cases in diffuse swelling, 16 cases in mixture, 8 cases in tumor, and 11 cases in elephantiasis. There was significant difference of serum TRAb levels among active stages in 6 variants (*F* = 3.379, *p* = 0.006). The highest levels of serum TRAb were in elephantiasis and the lowest were in nodule and tumor variants. The middle was in plaque, diffuse swelling, and mixture. In the 3 variants, diffuse swelling had bigger area of involvement and its level of serum TRAb was also higher. Because the elephantiasis variant had the biggest area or volume and the nodule variant had the smallest ones, so the data demonstrated that the bigger lesional area or volume was, the higher serum TRAb level was with activation of PTM lesions.

At stable stage, there were 17 cases. The mean value of TRAb level at each variant was lower than 40 IU/L and dramatically lower than that at active stage of corresponding variant.

At sclerotic stage, there were 2 cases including 1 case in diffuse swelling and 1 case in elephantiasis. Their serum TRAb levels were, respectively, lower than that at active stage of corresponding variant.

At receding stage, there were 12 cases including 4 cases in nodule, 6 cases in diffuse swelling, and 2 cases in elephantiasis. Their mean values of serum TRAb levels were, respectively, lower than those at active stage of corresponding variant.

The means of serum TRAb levels at stable, sclerotic, and receding stage between variants were not statistically analyzed because case numbers were too few.

### 3.8. Correlation of Histopathological Characteristics with Evolving Stages

Histopathologically, perivascular mononuclear cell infiltration was dramatic at active stage but a few or few at stable, sclerotic, and receding stages when HE stained.

**Figure 11:**
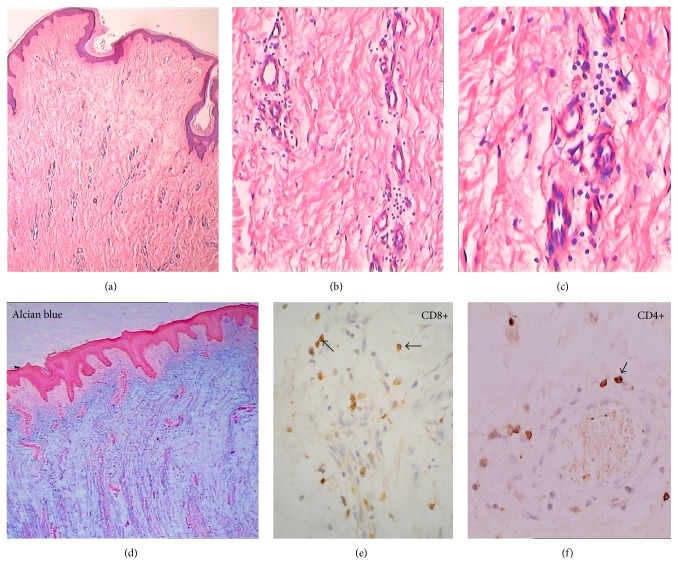
Large amounts of mucopolysaccharide deposits, numbers of newly formed capillaries, and a perivascular lymphocyte infiltrate at active stage of PTM. (a) H&E ×4. The epidermis was essentially normal. Just below the epidermis is a relatively unaltered strip of papillary dermis. The upper layer of reticulate dermis was obvious mucin degeneration with widely separated, frayed, and fragmented collagen bundles. In the lower layer of reticulate dermis were seen numbers of newly formed capillaries, a perivascular inflammatory cell infiltrate, and proliferation of fibroblasts. (b) H&E ×20 and (c) H&E ×40. Perivascular and dermal lymphocyte infiltrates were seen. (d) H&E ×4. Alcian blue staining showed large amounts of mucopolysaccharide deposits in the lower layer of papillary dermis and reticulate dermis. (e) ×40. Immunohistochemical staining by anti-CD8 monoclonal antibody showed a large proportion of infiltrates were CD8+ T lymphocytes (arrows) in the dermis and perivascular regions. (f) ×40. Immunohistochemical staining by anti-CD4 monoclonal antibody showed a CD4+ T-cell infiltrate in the dermis and perivascular regions.

80 cases were immunohistochemically stained with anti-CD4 monoclonal antibody. 63 cases were at active stage and 17 cases at stable stage. 90 cases were immunohistochemically stained with anti-CD8 monoclonal antibody. 73 cases were at active stage and 17 cases were at stable stage. 80% cases at active stage were positive stained with anti-CD4 antibody and 10% positive at stable stage. 100% cases at active stage were positive stained with anti-CD8 antibody and 33% positive at stable stage (Figure 11). These data showed that humoral and cell-mediated autoimmunity participated in the activation of PTM lesions.

### 3.9. Correlation of TRAb Levels with Remission and Relapse after Treatment at Our Department

After diagnosed at our department, 215 cases with PTM received therapy except 1 case subsiding spontaneously. 125 cases had complete records of serum TRAb levels before intralesional steroid therapy, in the process of therapy and remission after therapy. Comparisons of TRAb titers between before therapy and remission after therapy were shown in Table 5. The serum TRAb level after therapy was dramatically less than that before therapy (*p* = 0.000). However, after PTM lesions disappeared, 41.6% cases still had high titers of TRAb (>40 IU/L), 70.4% higher than 20 IU/L, and only one case in the normal value. In addition, there were 3 cases whose TRAb titers did not decrease but increased after remission and their lesions relapsed soon after. The case with normal TRAb level after remission was the nodule variant and without exophthalmos but other 124 cases were with exophthalmos.

**Figure 12:**
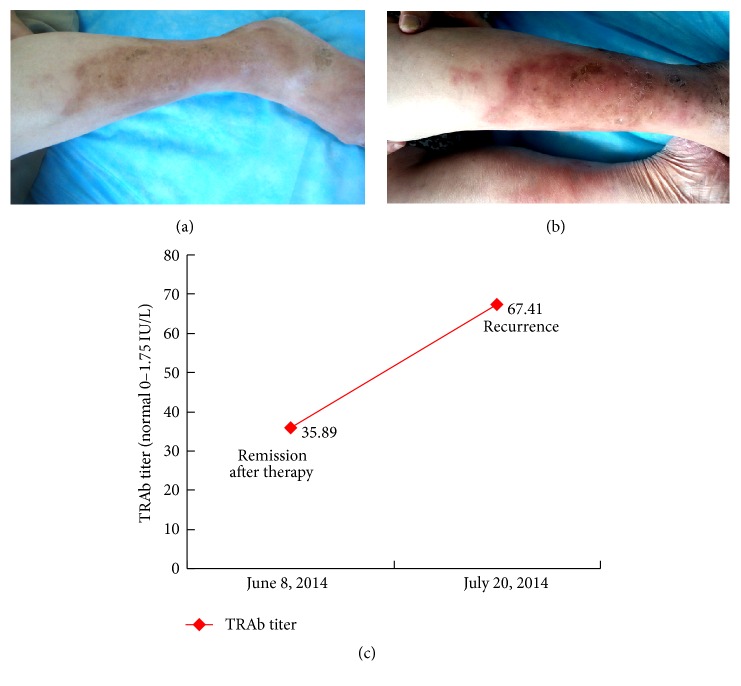
The serum TRAb level increased when the lesion recurred. (a) On June 8, 2014, the lesion on the left lower leg was at remission after therapy and pigmented. (b) On July 20, 2014, the lesions on both lower legs recurred and presented as erythematous, diffuse swelling. (c) The serum TRAb level was 35.89 IU/L when remission after therapy on June 8,2014 and increased to 67.41 IU/L when recurrence on July 20, 2014.

Following up with the 125 cases after complete response to intralesional steroid, 42 cases (33.6%) relapsed in 1.5 years. In the 42 cases, 25 cases had 1 relapse, 12 cases 2 relapses, 4 cases 3 relapses, and 1 case 4 relapses. Totally there were 65 relapses. The serum TRAb levels at relapse were dramatically higher than those at remission (*p* = 0.000). Compared with recurred cases, serum TRAb levels in nonrecurred cases were not only lower but also more stable (not dramatically fluctuating). Specifically, the serum TRAb levels in 67 nonrecurred cases were dramatically lower than those in 65 relapses of 42 recurred cases (*p* = 0.000). 16 cases in the nonrecurred group were lack of measuring serum TRAb levels in the follow-up. These data demonstrated that PTM relapsed when serum TRAb levels went up further on the basis of high TRAb titers (see Table 5 and Figure 12).

### 3.10. Clinical Outcomes of 6 Variants and Its Prognostication by Trends of Autoimmune Activity in Courses

**Figure 13:**
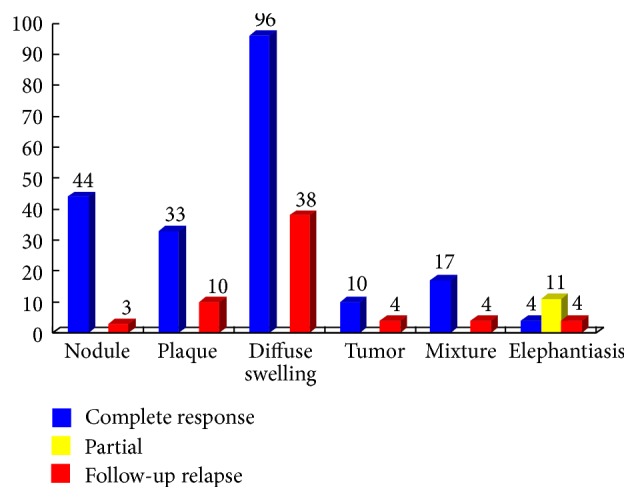
Clinical outcome of 215 cases with PTM after therapy and in the 1.5-year follow-up. After diagnosed at our department, 215 cases received therapy and underwent 1.5-year follow-up after therapy. The numbers above the color rectangles represented the case numbers of 6 variants, respectively. In 6 variants, blue rectangles indicated the case numbers of complete response to therapy; yellow rectangles showed the case numbers of partial response to therapy and red rectangles indicated the case numbers of relapse after therapy in the 1.5-year follow-up.

In 215 cases, 44 cases with nodule, 33 cases with plaque, and 96 cases with diffuse swelling were treated with treatment Protocol 1. 15 cases with elephantiasis were treated with Protocol 2. 10 cases with tumor and 17 cases with mixture were treated with Protocol 3. All cases treated with Protocols 1 and 3 obtained complete response. Four cases with elephantiasis had complete response but 11 cases had partial response. In the 1.5-year follow-up, 20 cases were lost. 195 cases finished the follow-up. One or more than one relapses occurred, respectively, in 3/30 cases with nodule, 10/33 cases with plaque, 38/96 cases with diffuse swelling, 8/27 cases with mixture or tumor, and 4/4 cases with elephantiasis (Figure 13).

**Figure 14:**
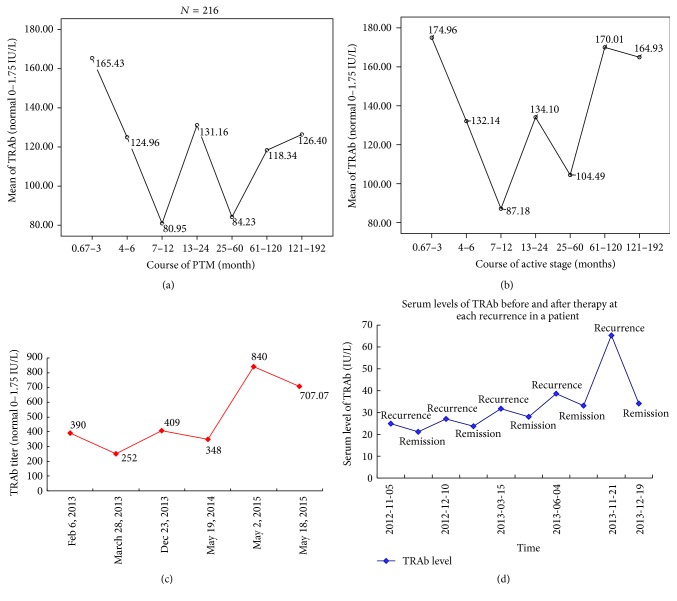
The fluctuation of serum TRAb levels with the extension of course. (a) Means of serum TRAb titers fluctuated with the extension of course in 216 PTM cases. The serum TRAb titers were 165.43 ± 187.162 IU/L (*N* = 33) between 20 days and 3 months of course, 124.96 ± 105.861 IU/L (*N* = 30) between 4 and 6 months, 80.95 ± 76.816 IU/L (*N* = 52) between 7 and 12 months, 131.16 ± 161.686 IU/L (*N* = 43) between 13 and 24 months, 84.23 ± 96.621 IU/L (*N* = 27) between 25 and 60 months, 118.34 ± 231.437 IU/L (*N* = 27) between 61 and 120 months, and 126.40 ± 153.059 IU/L (*N* = 4) between 121 and 192 months. By multiple comparisons with* Post Hoc* Tests, the serum TRAb titers between 0.67 and 3 months were statistically higher than those between 7 and 12 months (*p* = 0.011) and those between 25 and 60 months (*p* = 0.035), respectively. Mean difference and 95% confidence intervals were 84.48 [19.74, 149.22] and 81.2 [5.71, 156.69], respectively. There were no significant differences among 0.67–3, 4–6, 13–24, 61–120, and 121–192 months of PTM course (*p* > 0.05). The data demonstrated that the serum TRAb levels fluctuated in the 16-year course. (b) In 185 cases at active stage, means of serum TRAb titers fluctuated with the extension of a 192-month course. (c) In a PTM case with repeats of remission and relapse in about 2 years after a 14-year history, serum TRAb titers fluctuated between 252 IU/L and 840 IU/L. Even though his course was more than 14 years and steroid therapy, there were no trends of autoimmunity activity to stop and of PTM lesions to subside spontaneously. (d) In a patient with 5 repeats of recurrence and remission from November 5, 2012, to December 19, 2013, serum TRAb titers increased when lesion recurred and decreased when lesions subsided after therapy.

The period of 1.5-year follow-up was too short to find natural history and long-term outcome of PTM. In order to obtain the aim, the correlation of TRAb levels with the course of PTM was investigated in 216 cases with a chronic course. So the courses of 216 cases when diagnosed at our department were arbitrarily divided into 7 periods, which were 0.67 to 3, 4 to 6, 7 to 12, 13 to 24, 25 to 60, 61 to 120, and 121 to 192 months, respectively. The means of serum TRAb levels at each period were shown in Figure 14(a). The TRAb levels fluctuated with the extension of course. Serum TRAb levels of 185 cases at active stage in the 16-year course were showed in Figure 14(b). The two figures showed that autoimmune activity in PTM patients would fluctuate with the extension of their courses and predicted that autoimmune activities of 216 cases would not stop in quite a long extension of PTM course after 16 years, because even at the lowest point, the TRAb titer (80.9 IU/L) was the 46.2 times of the normal value (≤1.75 IU/L).

From longitudinal observation of an individual course, one case with a 14-year course had an average of 3 recurrences per year in recent 3 years and the fluctuation of TRAb titers was on the higher levels from 252 IU/L to 840 IU/L (see Figure 14(c)). Another case showed that serum TRAb levels fluctuated with the recurrences and remissions after repeated therapy in one year (Figure 14(d)). These data demonstrated that the autoimmune activity of PTM had no trend to stop in the future with the extension of its course.

**Figure 15:**
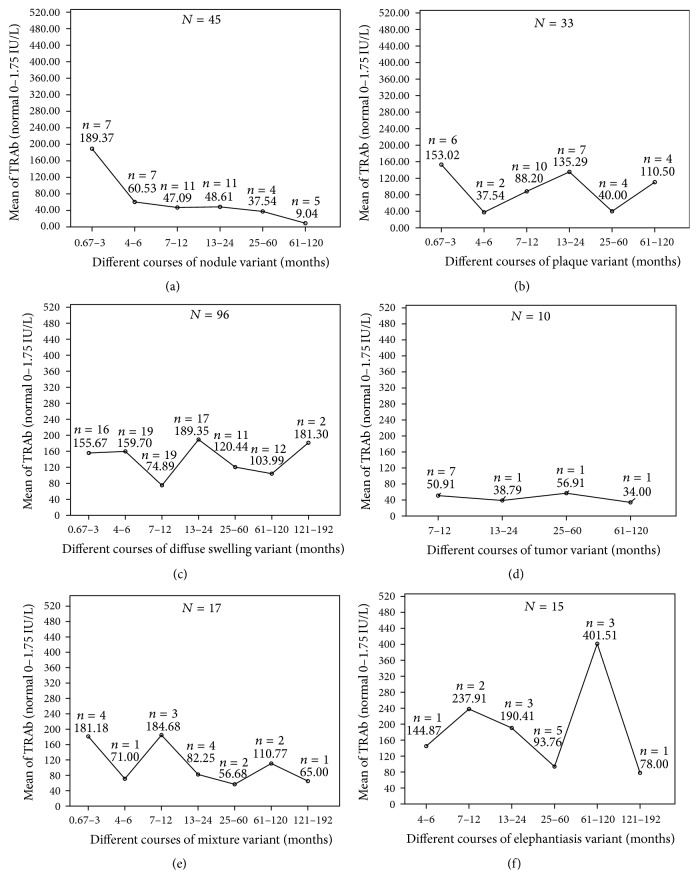
Trends of serum TRAb levels with the extension of their courses in 6 PTM variants of 216 cases. (a) In nodule variant, means of serum TRAb titers invariably decreased from 189.37 IU/L at 0.67–3 months to 9.04 IU/L at 61–120 months in the course. (b) In plaque variant, means of serum TRAb titers fluctuated with the extension of course. The lowest titers were 37.5 IU/L at 4–6 months and 40 IU/L at 25–60 months, respectively, and the highest titers were 153.02 IU/L at 0.67–3 months, 135.29 IU/L at 13–24 months, and 110.5 IU/L at 61–120 months, respectively. There was no decreasing trend of autoimmune activity with the extension of course. (c) In diffuse swelling variant, means of serum TRAb titers fluctuated with the extension of course. The lowest points were 74.89 IU/L at 7–12 months and 103.99 IU/L at 61–120 months. The highest points were 159.7 IU/L at 4–6 months, 189.35 IU/L at 13–24 months, and 181.3 IU/L at 121–192 months. (d) In tumor variant, means of serum TRAb titers fluctuated with the extension of course. (e) In mixture variant, means of serum TRAb titers fluctuated from 181.18 IU/L at 0.67–3 months to 65 IU/L at 121–192 months. (f) In elephantiasis variant, means of serum TRAb titers dramatically fluctuated with the extension of course. The lowest points were at 25–60 months and 121–192 months of course and the highest points were at 7–12 months and 61–120 months.

Through analyzing the correlation of TRAb level with the course of each variant, trends of serum TRAb levels with the extension of a 16-year course in each variant were showed in Figure 15. Serum TRAb levels of nodule variant decreased invariably from 189.37 IU/L to 9.04 IU/L. The curve showed that autoimmune activity of nodule variant had a trend to stop in the future with extension of course. However, serum TRAb levels of the plaque, diffuse swelling, tumor, mixture, and elephantiasis fluctuated on the higher levels with the extension of their courses. These curves showed that autoimmune activities of other 5 variants had no trends to stop in the future with the extension of course.

**Figure 16:**
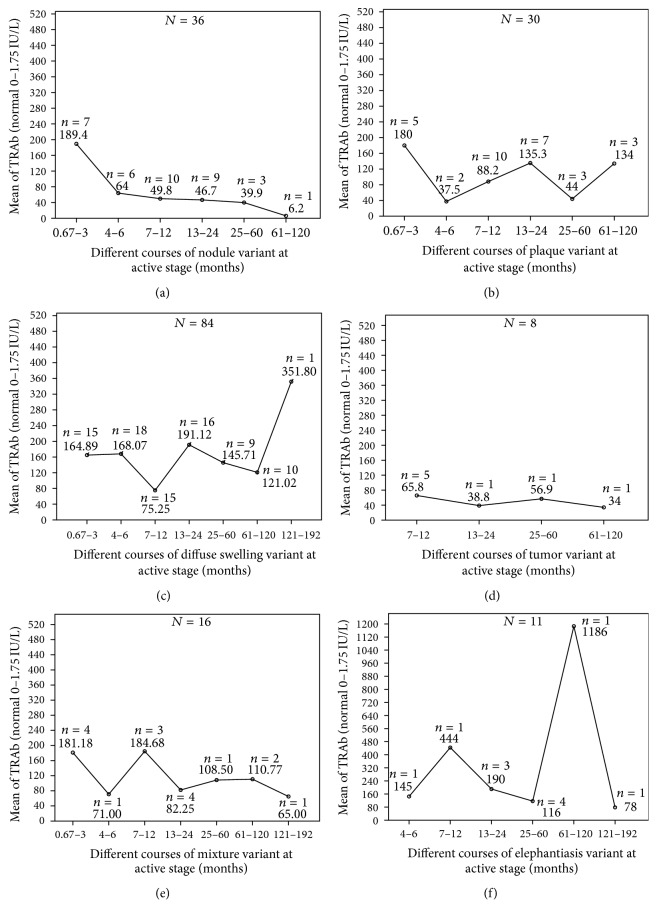
Trends of serum TRAb levels with the extension of courses at active stage in 6 PTM variants of 185 cases. (a) In nodule variant, means of serum TRAb titers at active stage invariably decreased from 189.4 IU/L at 0.67–3 months to 6.2 IU/L at 61–120 months in the course. (b) In plaque variant, means of serum TRAb titers at active stage went up-and-down with the extension of course. The lowest titers were 37.5 IU/L at 4–6 months and 44 IU/L at 25–60 months respectively and the highest titers were 180 IU/L at 0.67–3 months, 135.3 IU/L at 13–24 months and 134 IU/L at 61–120 months respectively. There was no decreasing trend of autoimmune activity with the extension of course. (c) In diffuse swelling variant, means of serum TRAb titers at active stage went upward from 165 IU/L at 0.67–3 months to 352 IU/L at 121–192 months with the extension of course. The trend of autoimmune activity fluctuated up with the extension of course. (d) In tumor variant, means of serum TRAb titers at active stage fluctuated with the extension of course. There was no difference of serum TRAb titers among 4 periods (*F* = 0.368, *p* = 0.781). (e) In mixture variant, means of serum TRAb titers at active stage fluctuated downward from 181.2 IU/L at 0.67–3 months to 65 IU/L at 121–192 months. (f) In elephantiasis variant, means of serum TRAb titers at active stage dramatically fluctuated with the extension of course (*F* = 36.228, *p* = 0.001). The lowest points were at 4–6 months, 25–60 months, and 121–192 months and the highest points were at 7–12 months and 61–120 months.

At active stage, total cases were 185, excluding 17 cases at stable stage, 2 cases at sclerotic stage, and 12 cases at receding stage. There were 36 cases with nodule, 30 cases with plaque, 84 cases with diffuse swelling, 16 cases with mixture, 8 cases with tumor, and 11 cases with elephantiasis, respectively. Trends of serum TRAb levels with the extension of course in 6 variants were showed in Figure 16. Excluding influencing factors of stable, sclerotic, and receding stages, similar curves were still acquired. These curves corresponded with the clinical outcomes of PTM.

## 4. Discussion

PTM occurs in 1.6% of patients with thyroid diseases, 1.7% of patients with Grave's thyrotoxicosis [5], and 15% of patients with severe Graves' ophthalmopathy [21]. 97% of patients with PTM are associated with Graves' ophthalmopathy [21] and 20% associated with acropachy [4]. Thyroid dysfunction, like ophthalmopathy and acropachy, is not the cause of PTM but they are overlapping conditions. They together belong to a syndrome and have an autoimmune pathogenesis in common. PTM often occurs in farming occupation probably because traditional cultivating method in West China makes legs more easily to be injured. Symptoms of PTM include different variants of lesions, local pruritus, hyperhidrosis, hypertrichosis, and orange peel-like appearance, occasionally prickling.

In our case series, we first found that clinically active, stable, sclerotic, and receding stages existed in the chronic course of PTM. The six variants of PTM evolved from first appearing lesions through intermittent periods of activation and stability or remission and relapse. The process was caused by the fluctuation of autoimmune activity, which was indicated by serum TRAb levels. So serum TRAb levels could be as an indicator that reflected not only the fluctuation of autoimmune activity but also clinical change of lesions. TRAb levels increased at activation and relapse of PTM. Its level decreased at stability and remission. At active stage, the increase of TRAb levels and perivascular CD4+ and CD8+ T lymphocyte infiltration implied the activation of humoral and cell-mediated autoimmunity, which would result in excessive hyaluronan deposits and fibrous tissue hyperplasia. The high viscosity of hyaluronan in solution made an equivalent weight of hyaluronate occupy 75,000 times the volume of collagen [22, 23]. Hyaluronan in tissue could hold large quantities of water. So excessive hyaluronan deposits caused lesions of PTM to enlarge and protrude rapidly over the skin. That was why we could find clinically that swelling of lesions mitigated in the morning or after getting up and aggravated in the evening or after long-time standing. Proliferated fibroblasts, together with its synthesized hyaluronan and collagen fibers, infiltrated subcutaneous tissue and its peripheral skin [19]. At stable stage, the inflammation and infiltration abated or stopped because humoral and cell-mediated autoimmune activity decreased. The synthesis of hyaluronan also decreased and previous deposited hyaluronan was rapidly metabolized but proliferated fibrous connective tissue still remained. As a result, repeated intermittent periods of active and stable stages made first appearing lesions worsen intermittently. Finally, they evolved to the six variants. At sclerotic stage, serum TRAb level was lower than that of active stage. After repeats of active stages, autoimmune activity decreased and TRAb persisted on the lower level. Proliferated fibrous connective tissue remained in lesions with remarkably reduced amounts of hyaluronan. The thickening dermis entered sclerotic stage and became hardening. They might persist in sclerotic stage if serum TRAb level was stable on the lower level or entered the active stage again when serum TRAb level went up. Afterwards, the lesions did not recede into the normal appearance of skin without therapy. When serum TRAb level decreased persistently without excessive hyperplasia of fibrous tissue, the lesions would subside spontaneously. So did the nodule variant.

In addition to the stages in its evolving process, the lesions of PTM are also characterized by morphological diversity, inflammation, infiltration, and hyperplasia. Cairns described three clinical appearances of localized form, diffuse nonpitting edema, and elephantiasis [14]. Isolated cases in PubMed database reported nodule, plaque, tumor, and elephantiasic variants [24–27]. Some large case series reported more variants [5, 17, 21, 28, 29]. In the largest case series, the morphology of PTM consisted of 6 variants and its subvariants. Except 6 subvariants of the plaque variant, there were also the polypoid and verrucous variants reported [17, 18]. The morphological diversity of PTM lesions was correlated with different intensity of autoimmune activity, different kinds, and frequencies of local injury and different stages of lesion-evolving process.

Variants are correlated with different intensity of autoimmune activity. The subvariants are mainly caused by different kinds of local injury. Local injury includes open wound, close wound, and itching. In 30.1% cases of the study, we found local injury (trauma) happened before PTM occurrence. Trauma repeating or occurring at multiple sites resulted in occurrence of multiple new-formed lesions at multiple sites on the surface of PTM lesions. PTM occurs not only at the normal skin but also at the traumatic scar. Of particular interest are previous scars formed many years ago which can be reactivated to grow and puff up like keloid but not keloid when PTM is active. Local trauma has been known as a precipitating factor of PTM and it makes stable lesions to be active or disappearing lesions to relapse [30, 31]. So local injury is the main cause of subvariants at each variant but not of variants.

The inflammatory lesion presented as erythema and lymphocyte infiltration. The infiltration characteristics of PTM meant that lymphocytes penetrated lesions of PTM and the fibrous connective tissue with excessive hyaluronan deposits and fibroblast proliferation infiltrated subcutaneous tissue and surrounding normal skin [18, 19]. The hyperplasia of PTM manifested as hyperkeratosis and acanthosis of epidermis, dermal thickening, and hypertrichosis [18]. The phenomena were initiated by autoimmune reactions (humoral and cell-mediated immunity) to autoantigens (mainly TSHR) on the fibroblasts [32, 33], fibrocytes [34], and mast cells [33].

The role of TRAb in the pathogenesis of PTM had been controversial since Kriss JP first found the effect of TRAb on PTM in 1964 [35–37]. Recently, Shih et al. [38] found that TRAb level correlated positively and significantly with skin thickness change. In our case series, serum TRAb levels were found to be positively correlated with severity of PTM lesions (variants). Through comparing serum TRAb levels between active and stable stage, before and after therapy, remission and relapse, and recurred cases and nonrecurred cases, TRAb was found to play an important role in the initiation and development of PTM lesions. However, in most PTM patients with ophthalmopathy, when PTM lesions disappeared after therapy, TRAb levels decreased but were still higher than normal level. Even in a few cases, TRAb levels didnot decrease but increased. The phenomenon hinted that ophthalmopathy or hyperthyroidism was active or severe because serum TRAb levels came from the contributions of not only PTM but also Graves' ophthalmopathy and autoimmune thyroid diseases [39, 40].

On the one hand, there was an overlapping of absolute TRAb levels between active stage and stable stage or remission and relapse in different individuals. And there were no differences in serum TRAb positive rates of Grave's patients between with PTM and without PTM. On the other hand, the fluctuation of autoimmune activity in the same individual was correlated with repeated intermittent periods of activation and stability or remission and relapse. And serum TRAb levels in recurred cases were statistically higher than those in nonrecurred cases. The phenomena implied that susceptible genes may also exist in PTM individuals and maybe multifactors take part in the pathogenesis of PTM. These factors include genetic susceptibility, humoral and cell-mediated autoimmunity, environment, and trauma. The interaction of TRAb and T lymphocytes with TSHRs on the fibroblasts, fibrocytes, and mast cells [41, 42] may play a central role in excessive synthesis of hyaluronan and hyperplasia of fibrous connective tissue.

As to outcome of PTM, generally accepted view was that it was self-limited and lesions usually resolved spontaneously without requirement of therapy [7, 8]. This view was contradicted to the clinical reality. Actually, most cases of PTM underwent a chronic process with repeated intermittent periods of active and stable stages after occurrence, only nodule subsiding spontaneously. Through analyzing the correlation of TRAb level with the course of PTM, the results indicated that autoimmune activity in nodule variant gradually decreased with the extension of course and would go to stop in the future, but in other 5 variants the autoimmune activities always fluctuated on higher level with the extension of courses and would not stop in the future. Nodule variant clinically subsides spontaneously and is self-limited but the other 5 variants arenot. Therefore, according to the curve of TRAb titer changes in the course of PTM, we can predict that the plaque, diffuse swelling, tumor, mixture, and elephantiasis undergo a chronic process of waving aggravation with the clinical activation and stability and the fluctuation of autoimmune activity, nodule variant finally subsiding spontaneously.

In the series, 79.2% (5 variants) were moderate to severe and required therapy. Even receiving therapy, some cases still relapsed. Severe variants, especially elephantiasis, were refractory to intralesional steroid maybe because of large amounts of fibrous connective tissue and persistence of intensive autoimmunity. The tumor and elephantiasis resulted in both disfiguration and disability. So PTM requires early treatment to avoid severe variants [19, 43].

## 5. Conclusions

PTM is a rare autoimmune dermopathy overlapped with autoimmune dysthyroidism, thyroid-associated ophthalmopathy, and acropachy. Under the susceptible background, excessive synthesis of hyaluronan and hyperplasia of fibrous connective tissue in PTM lesions are initiated and promoted by humoral and cell-mediated autoimmunity to TSHRs on the fibroblasts, fibrocytes, and mast cells in the dermis. In the chronic course, morphological diversity (variants and subvariants) of PTM is caused by different intensity of autoimmune activity, different stages of lesion-evolving process, and local injury. Clinical outcomes of six variants are determined by trends of autoimmune activity with the extension of course. Mild nodule variant can disappear spontaneously and it is self-limited while moderate to severe variants including plaque, diffuse swelling, tumor, mixture, and elephantiasis worsen intermittently and they are not self-limited. Early intralesional steroid treatment of PTM can prevent severe variants from occurring.

## Figures and Tables

**Table 1:** Demographic and dysthyroid-associating characteristics of 6 variants when diagnosed at our department.

Variables	Total	Nodule	Plaque	Diffuse swelling	Mixture	Tumor	Elephantiasis	Statistical test	*p* value
Patient number	216	45	33	96	17	10	15		

Sex									
Female (number)	120	17	18	62	12	4	7	*χ* ^2^ = 11.958	0.035
Male (number)	96	28	15	34	5	6	8		

Age									
*X* ± SD year	46.7 ± 12.2	42.8 ± 9.6	46.3 ± 14.4	48.2 ± 12.3	46.7 ± 10.1	39.8 ± 10.0	53.6 ± 12.9	*F* = 2.956	0.013
Range (min–max) year	17–80	21–65	17–80	21–80	25–60	18–50	26–79		

Occupation									
Chinese farmer	144	27	17	68	14	10	8	*χ* ^2^ = 31.955	0.044
City dweller	57	13	11	26	2		5		
Worker	10	2	4	1	1		2		
Cadre	4	3		1					
Teacher	1		1						

Dysthyroid-associated									
Graves	215	45	33	96	16	10	15	*χ* ^2^ = 11.76	0.038
Hashimoto thyroiditis	1				1				
Exophthalmos	206	35	31	89	12	9	14	*χ* ^2^ = 1.503	0.913
Acropachy	28	7	10	14	3	3	5	*χ* ^2^ = 13.413	0.02

Temporal relationship between PTM onset and dysthyroid diagnosis									
Before dysthyroid	4	3					1	*χ* ^2^ = 20.184	0.028
Simultaneous	30	9	6	11			4		
After dysthyroid	182	33	27	85	17	10	10		

Dysthyroid course									
*X* ± SD month	50.4 ± 45.1	41.4 ± 42.6	45.3 ± 41.8	48.3 ± 41.1	67.4 ± 57.2	66.0 ± 58.3	66.5 ± 51.0	*F* = 1.537	0.18
Range (min–max) month	5–204	6–192	5–180	5–192	7–192	24–204	9–168		

**Table 2:** Clinical manifestations of 6 variants when and before diagnosed at our department.

Variables	Total	Nodule	Plaque	Diffuse swelling	Mixture	Tumor	Elephantiasis	Statistical test	*p* value
Patient number	216	45	33	96	17	10	15		

PTM course									
*X* ± SD month	27.6 ± 37.6	24.48 ± 29.06	25.3 ± 31.8	26.8 ± 36.2	30.4 ± 38.9	22.2 ± 20.8	48.0 ± 45.2	*F* = 1.231	0.296
Range (min–max) month	0.67–192	0.67–120	1–120	1–192	1–144	12–72	6–180		

Symptom									
Pruritus (number)	116/216 (53.7%)	25	16	50	8	4	13	*χ* ^2^ = 8.137	0.149
Hyperhidrosis (number)	97/216 (44.91%)	8	10	42	15	8	14	*χ* ^2^ = 48.379	0.000
Hypertrichosis (number)	65/216 (30.09%)	10	12	28	5	3	7	*χ* ^2^ = 3.944	0.558
Orange peel-like appearance (number)	108/216 (50%)	14	23	49	9	5	8	*χ* ^2^ = 11.711	0.039

Local injury history									
Yes (number)	65	12	7	25	7	4	10	*χ* ^2^ = 13.234	0.021
No (number)	151	33	26	71	10	6	5		

Scars on lesions									
Yes (number)	104	14	15	49	9	5	12	*χ* ^2^ = 11.915	0.036
No (number)	112	31	18	47	8	5	3		

PTM relapse									
Yes (number)	72	16	9	35	6	3	3	*χ* ^2^ = 2.347	0.799
No (number)	144	29	24	61	11	7	12		

Stages									
Active	185	36	30	84	16	8	11	*χ* ^2^ = 16.543	0.347
Stable	17	5	3	5	1	2	1		
Scerotic	2			1			1		
Receding	12	4		6			2		

**Table 3:** 6 variants evolved from first appearing lesions at the onset of PTM.

Variables	Total	Nodule	Plaque	Diffuse swelling	Mixture	Tumor	Elephantiasis	Statistical test	*p* value
Patient number	216	45	33	96	17	10	15		

PTM course									
*X* ± SD month	27.6 ± 37.6	24.48 ± 29.06	25.3 ± 31.8	26.8 ± 36.2	30.4 ± 38.9	22.2 ± 20.8	48.0 ± 45.2	*F* = 1.231	0.296
Range (min–max) month	0.67–192	0.67–120	1–120	1–192	1–144	12–72	6–180		

First appeared lesions									
Nodule (number)	118	45	27	22	10	10	4	*χ* ^2^ = 108.393	0.000
Swelling erythema (number)	96		6	74	6	1	9		
Papule (number)	2				1		1		

**Table 4:** Serum TRAb levels at 4 stages of 6 variants at diagnosis of PTM.

Variables	Total	Nodule	Plaque	Diffuse swelling	Mixture	Tumor	Elephantiasis	Statistical test	*p* value
Patient number	216	45	33	96	17	10	15		

TRAb									
*X* ± SD (IU/L)	115.89 ± 148.41	66.61 ± 121.70	103.77 ± 102.79	136.48 ± 142.32	122.27 ± 129.47	48.61 ± 31.26	196.22 ± 298.39	*F* = 2.817	0.017
min–max (IU/L)	2.04–1186.20	2.04–827	18.07–418.66	6.22–789.18	4.86–525.21	12–103	3.11–1186.20		

Active stage									
Number	185	36	30	84	16	8	11		
TRAb (*X* ± SD IU/L)	130.12 ± 155.08^a^	76.48 ± 134.17	111.27 ± 104.93	149.51 ± 146.08	129.61 ± 130.01	57.32 ± 28.67	262.72 ± 326.12	*F* = 3.379	0.006
min–max (IU/L)	5–1186.20	5–827	21.71–418.66	19.9–789.18	29.74–525.21	26.64–103.00	39.08–1186.2		

Stable stage									
Number	17	5	3	5	1	2	1		
TRAb (*X* ± SD IU/L)	17.64 ± 18.14	25.28 ± 29.98	28.69 ± 10.98	10.40 ± 4.92	4.86	13.76 ± 2.49	3.11		
min–max (IU/L)	2.04–75	2.04–75	18.07–40.00	6.22–18.63	4.86–4.86	12–15.52	3.11–3.11		

Sclerotic stage									
Number	2			1			1		
TRAb (*X* ± SD IU/L)	80.09 ± 68.19			128.3			31.87		
min–max (IU/L)	31.87–128.3			128.3			31.87		

Receding stage									
Number	12	4		6			2		
TRAb (*X* ± SD IU/L)	41.59 ± 52.16	29.44 ± 12.13		60.5 ± 70.19			9.17 ± 3.22		
min–max (IU/L)	6.89–161.00	18–40		7.3–161.00			6.89–11.44		

a: TRAb levels among active, stable, sclerotic, and receding, *F* = 4.279, *p* = 0.006.

**Table 5:** Comparison of TRAb titers between before and after therapy, remission and relapse, and recurred and nonrecurred cases.

TRAb variables	Group 1	Group 2	Statistical test	*p* value
	Before therapy	After therapy		

Number	125	125		
Mean (*X* ± SD IU/L)	140.83 ± 182.12	73.55 ± 94.51	Paired test *t* = 5.21	0.000
Range (min-max IU/L)	1.87–1186.2	1.13–585.69		
TRAb titer distribution				
<20 IU/L (no.)	12	37	Pearson chi-square *χ* ^2^ = 19.225	0.000
20–40 IU/L (no.)	34	36		
40–100 IU/L (no.)	27	22		
>100 IU/L (no.)	52	30		

	Remission	Relapse		

Number (65 times of 42 cases)	65	65		
Mean (*X* ± SD IU/L)	71.454 ± 78.473	132.146 ± 162.803	Paired test *t* = 4.876	0.000
Range (min–max IU/L)	9.4–351.8	19.8–840		

	Nonrecurred cases	Recurred cases		

No.	67	65		
Mean (*X* ± SD IU/L)	22.76 ± 14.577	132.146 ± 162.803	Independent test *t* = 5.477	0.000
Range (min–max IU/L)	0.5–60.8	19.8–840		
